# Regulatory Networks, Management Approaches, and Emerging Treatments of Nonalcoholic Fatty Liver Disease

**DOI:** 10.1155/2022/6799414

**Published:** 2022-11-08

**Authors:** Bing Yang, Xi Yang, Xumei Tan, Liqing Lu, Wei Fan, Lucía Barbier-Torres, Justin Steggerda, Ting Liu, Heping Yang

**Affiliations:** ^1^Department of Geriatric Endocrinology and Metabolism, Guangxi Key Laboratory of Precision Medicine in Cardio-Cerebrovascular Diseases Control and Prevention, Guangxi Clinical Research Center for Cardio-Cerebrovascular Diseases, The First Affiliated Hospital of Guangxi Medical University, Nanning, China; ^2^Department of Gastroenterology and Thoracic Surgery, Key Laboratory of Cancer Proteomics of Chinese Ministry of Health, Xiangya Hospital, Central South University, Changsha, Hunan 410008, China; ^3^Division of Digestive and Liver Diseases, Cedars-Sinai Medical Center, Los Angeles, CA 90048, USA; ^4^Northwestern Medicine Organ Transplantation Center, Arkes Pavilion, Chicago, IL 60611, USA

## Abstract

The pathogenesis of NAFLD is complex and diverse, involving multiple signaling pathways and cytokines from various organs. Hepatokines, stellakines, adipokines, and myokines secreted by hepatocytes, hepatic stellate cells, adipose tissue, and myocytes play an important role in the occurrence and development of nonalcoholic fatty liver disease (NAFLD). The nuclear factor kappa-light-chain-enhancer of activated B cells (NF-*κ*B) contributes to the progression of NAFLD by mediating liver inflammation, immune response, hepatocyte death, and later compensatory proliferation. In this review, we first discuss the crosstalk and interaction between hepatokines, stellakines, adipokines, and myokines and NF-*κ*B in NAFLD. The characterization of the crosstalk of NF-*κ*B with these factors will provide a better understanding of the molecular mechanisms involved in the progression of NAFLD. In addition, we examine new expert management opinions for NAFLD and explore the therapeutic potential of silymarin in NAFLD/NASH.

## 1. Introduction

Nonalcoholic fatty liver disease (NAFLD) refers to hepatic steatosis >5% without excessive drinking, hepatitis, drug use, etc. [1]. NAFLD comprises a continuum of diseases ranging from simple hepatic steatosis to nonalcoholic liver disease (NASH), liver cirrhosis, and HCC [2]. At present, the global prevalence of NAFLD is increasing, posing a serious threat to human life and health, and there is an urgent need for new methods to prevent and treat NAFLD. Metabolic syndrome, which typically includes abdominal obesity, hyperglycemia, dyslipidemia, and systemic hypertension, is the strongest risk factor for NAFL and NASH, and the association is bidirectional [3].

The nuclear factor kappa-light-chain-enhancer of activated B cells (NF-*κ*B) transcription factor family is composed of five cellular DNA-binding subunits: p50, p52, cRel, p65 (also known as RELA), and RELB, which are encoded by *NF-κB1*, *NF-κB2*, *REL*, *RELA*, and *RELB*, respectively. The heterodimer, p50/p65, is the most common form of NF-*κ*B and is a key driver in liver cancer [4]. NF-*κ*B is an important inflammatory mediator, involved in both the inflammatory pathway and lipid metabolism. NF-*κ*B promotes the progression of NAFLD by mediating liver inflammation, immune response, hepatocyte death, and later compensatory proliferation [5]. While basal NF-*κ*B activity in hepatocytes can promote HCC by inducing inflammation, it also can suppress the compensatory proliferation and prevent HCC development by inhibiting apoptotic pathways [5,6]. Considering this manner, we can say that NF-*κ*B has a dual function in the progression of NAFLD to HCC.

Recent studies have found that hepatokines, myokines, adipokines, and stellakines are also involved in the regulation of the pathogenesis of NAFLD. The characterization of the crosstalk of NF-*κ*B with these factors will contribute to a better understanding of the molecular mechanisms involved in the progression of NAFLD. In this review, we describe the relationship between NF-*κ*B and hepatokines, stellakines, adipokines, and myokines in hepatic stellate cells (HSCs), adipocytes, hepatocytes, and myocytes, respectively, and their regulatory signaling pathway. We then reviewed recent Wenxian counties to summarize new screening, diagnosis, and referral modalities for NAFLD/NASH, as well as potential therapeutic agents.

## 2. Hepatokines and NF-*κ*B Activation

**Figure 1:**
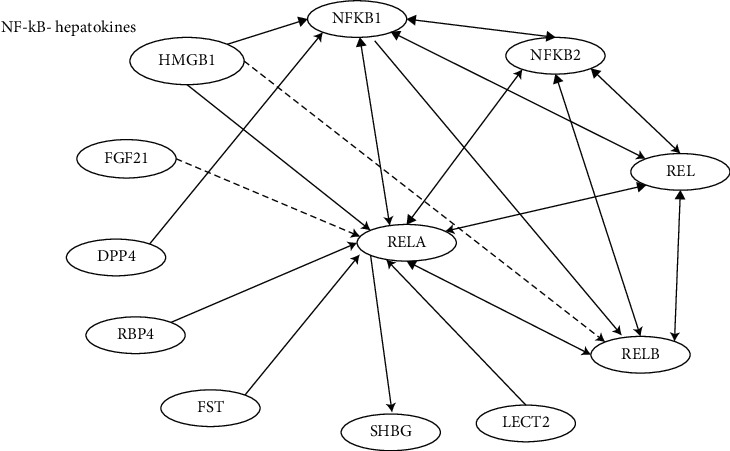
NF-*κ*B and hepatokines. IPA showed the molecular pathways of hepatokines and NF-*κ*B network. FGF21: fibroblast growth factor 21, HMGB1: high-mobility group box 1, DPP4: dipeptidyl peptidase 4, RBP4: binding protein 4, ASHG: alpha 2-HS glycoprotein (or FETUA), SHBG: sex hormone binding globulin, LECT2: leukocyte cell-derived chemotaxin 2, FST: follistatin. Solid lines indicate direct regulation; dashed lines indicate indirect regulation.

As the primary parenchymal cell type in the liver, hepatocytes may suffer injury or death under various pathogenic conditions accompanying metabolic changes, such as obesity, insulin resistance (IR), and diabetes. Under these circumstances, hepatocytes secrete signaling proteins, named hepatokines, which are closely related to lipid metabolism, oxidative stress, IR, inflammation, and other pathophysiological processes [7]. Studies have shown that NF-*κ*B plays an important role in the initiation and development of NAFLD. Knocking down p65 in mice on a high-fat diet (HFD) reduces liver steatosis and IR [8]. IKK-*β* acts as inhibitor of nuclear factor kappa-B kinase subunit beta. The mouse model of constitutively active IKK-*β* in hepatocytes leads to activated NF-*κ*B independently of inflammation and promotes liver fat synthesis and cholesterol synthesis [9]. Under normal circumstances, NF-*κ*B is isolated in the cytoplasm bound to the *κ*B inhibitor (I*κ*B) protein, which inhibits its nuclear localization. Site-specific phosphorylation of I*κ*B (*α*, *β*, and *ε*) by IKK-*β* plays an important role in the activation of NF-*κ*B [10]. In NAFLD, increased adipogenesis and mitochondrial dysfunction lead to oxidative stress, which can inhibit insulin signaling by activating IKK-*β* and Jun N-terminal kinase (JNK) [11,12]. Hepatokines can also regulate the expression of IKK-*β* [13]. The latter inhibits insulin signal transduction by phosphorylating insulin receptor substrates 1 and 2 [14]. This suggests that IKK-*β* deficiency improves glucose tolerance and insulin sensitivity and inhibits the NF-*κ*B pathway to limit adipogenesis and inflammatory processes. Hepatokines, including retinol fibroblast growth factor 21 (FGF21), high-mobility group box 1 (HMGB1), dipeptidyl peptidase 4 (DPP4), binding protein 4 (RBP4), alpha 2-HS glycoprotein (ASHG or FETUA), sex hormone binding globulin (SHBG), leukocyte cell-derived chemotaxin 2 (LECT2), and follistatin (FST), are involved in driving NAFLD by interaction with NF-*κ*B (Figure 1).

### 2.1. FGF21

FGF21 is an endocrine factor, mainly secreted by hepatocytes, which regulates the metabolism of liver, skeletal muscle, fat, and other organs. Studies have shown that FGF21 deficiency leads to glucose metabolism dysfunction as well as IR in mice, and exogenous FGF21 intake attenuates hepatic steatosis in mice [15]. However, other authors have suggested that serum FGF21 levels are elevated in patients with NAFLD and positively correlate with intrahepatic triglycerides (TG) [16]. “FGF21 resistance” may explain these seemingly opposite findings. *β*-Klotho is a single-pass transmembrane protein known as a co-receptor for FGF21 and is expressed mainly in liver. It is reported that TNF-*α* can inhibit the expression of FGF21 receptor *β*-klotho [17], which may reduce the response to FGF21 and result in increased pathological compensation to FGF21. In addition, NAFLD-related states such as insulin resistance and oxidative stress may also stimulate FGF21 expression. Farnesoid X (FXR) receptor is a ligand-activated transcription factor that regulates bile acid, glucose, and lipid metabolism. PPAR*α* acts as the master regulator of lipid metabolism. Regulatory properties of FGF21 derive from its intrinsic ability to act as ligand for a range of receptors including nuclear receptors FXR and PPAR*α* [18]. In parallel, FGF21 and NF-*κ*B crosstalk regulate the phenotype of HSCs and the progression toward NASH [19]. TLR4 is a cell surface pattern recognition receptor. IL-17A regulates the activities of NF-*κ*B and mitogen-activated protein kinases. The expression of NF-*κ*B is increased in FGF21 knockdown hepatocytes and promotes the progression of NASH to HCC through the axis of Toll-like receptor 4 (TLR4)/NF-*κ*B/IL-17A axis) [20]. FGF21 downregulates I*κ*B*α* phosphorylation and NF-*κ*B nuclear translocation in HSCs to inhibit their activation, reducing the ratio of Bcl-2 to Bax to promote apoptosis, thereby mitigating the deposition of *α*-smooth muscle actin (*α*-SMA) and collagen to reduce fibrosis [19]. In addition, studies have shown that NF-*κ*B negatively regulates FGF21 transcription by directly binding to its promoter DNA, and FGF21 can promote the transition of NAFLD to HCC through the NF-*κ*B pathway [21]. In NASH-HCC models, lack of FGF21 caused significant upregulation of the hepatocyte-derived IL-17A via TLR4 and NF-*κ*B signaling, which suggests that FGF21 and NF-*κ*B may be involved in the transition of NASH-HCC. Therapeutic approaches targeting FGF21 are expected to delay the progression of the disease.

### 2.2. HMGB1

HMGB1 is a highly conserved, abundant, nonhistone nuclear protein essential for life, which can regulate gene transcription and maintain nucleosome function. In addition, in liver injury such as NAFLD, HMGB1 can also be released by damaged hepatocytes due to excessive lipid accumulation [22]. After its release, HMGB1 binds to the receptor for advanced glycosylation end products as well as TLR receptors, subsequently activating a series of signaling cascades, such as NF-*κ*B and MAPK pathways, which further triggers liver inflammation and promotes the progression of the disease [23]. Studies have shown that HMGB1 is elevated in NAFLD patients and that it accelerates liver damage and inflammation in the early stages of NAFLD [24]. Deletion of HMGB1 in hepatocytes increases early body weight gain and lipid accumulation in mice and increases ER stress in liver and hepatocytes to promote liver injury during NAFLD [25]. Inhibition of HMGB1 release from hepatocytes attenuates liver damage [26]. Further mechanistic studies showed that HMGB1 is mainly involved in the progression of the disease by regulating NAFLD-related inflammation and fatty acid metabolism. We mentioned earlier that extracellular HMGB1 can promote the occurrence of sterile inflammation through NF-*κ*B. IKB-*α* is one of the binding targets of HMGB1, IKB-*α* acts as an inhibitor on NF-*κ*B, and deletion of HMGB1 downregulates NF-*κ*B/p65 phosphorylation and expression [27]. Studies have shown that enhancement of NF-*κ*B signaling by HMGB1 promotes NAFLD progression to HCC and regulates cell proliferation, invasion, and metastasis in HCC cell lines [28, 29]. Inhibiting this pathway can reduce oxidative stress in NASH mice and enhance the recruitment of inflammatory factors to alleviate liver injury [30]. Therefore, intrahepatic and extracellular HMGB1 play distinct roles, and blocking the HMGB1-NF-*κ*B axis and HMGB1 translocation may serve as a potential therapeutic target for reducing inflammation and NAFLD.

### 2.3. DPP4

DPP4 is involved in the pathological process of various chronic liver diseases, such as NAFLD, liver fibrosis, and liver cancer [31–33]. DPP4 is a ubiquitously expressed transmembrane protein that reduces the expression level of glucagon-like peptide 1 and regulates NAFLD through autocrine and paracrine effects on hepatic insulin signaling [34]. Soluble DPP4 activates MAPK and NF-*κ*B signaling cascades, enhances inflammatory responses in diseases, and exhibits strong pro-fibrotic functions in kidney-, lung-, and liver-related diseases. Based on this, DPP4 inhibitors, such as sitagliptin and alogliptin, which have been widely used in the treatment of T2DM, are being explored in liver disease. The nuclear factor erythroid 2–related factor 2 (Nrf2) is a major regulator of antioxidant and cellular protective genes [35]. Sitagliptin can directly inhibit the proliferation of activated HSCs and protect the liver by promoting Nrf2 and inhibiting NF-*κ*B pathway, reducing the levels of inflammatory factors such as TNF-*α* and pro-fibrotic factors such as TGF- *β* [36]. On the other hand, an ameliorative action of sitagliptin in NAFLD was demonstrated via decreasing HMGB1-mediated TLR4/NF-*κ*B signaling, which suppressed inflammation and reduced IR [31]. Alogliptin treatment can also inhibit the activation of HSCs and the deposition of alpha-smooth muscle actin (*α*-SMA) and extracellular matrix (ECM), alleviating NASH-related fibrogenesis [37]. Therefore, DDP4 is a potential target for the treatment of NAFLD and NAFLD-related liver fibrosis.

### 2.4. RBP4

Retinol binding protein 4 (RBP4) is both a hepatokine and an adipokine. Adipocyte-specific release of human RBP4 has been reported to promote hepatic steatosis [38]. A longitudinal study suggested that RBP4 levels are associated with the development and regression of NAFLD and that they are also an independent predictor of NAFLD progression [39]. It was shown that phosphorylation of RBP4 at Ser536 resulted in a marked increase in the activation of the NF-*κ*B subunit p65 [40]. In this work, it was also found that retinol-bound RBP4 significantly increased the nuclear content of the NF-*κ*B subunit p65 protein and its DNA-binding activity [40]. Both in human retinal capillary endothelial cells and human umbilical vein endothelial cells (HUVEC), RBP4-induced activation of NF-*κ*B and NADPH oxidase stimulates pro-inflammatory proteins expression, thereby inducing endothelial inflammation [40]. Furthermore, RBP4 could exert pro-inflammatory effects by activating TLR4 and c-JNK signaling in macrophages [41,42]. Studies have demonstrated that TLR4 is expressed in endothelial cells, and activation of downstream NF-*κ*B signaling through TLR4 signaling may become a reality [41, 42]. These results suggest that RBP4 may mediate NF-*κ*B-dependent inflammation by activating TLR4 signaling which may influence NAFLD disease progression.

### 2.5. FST

FST is a glycosylated plasma protein and a natural antagonist of activin A, which binds and inactivates members of the TGF-*β* family [43]. Five follistatin-like proteins have now been identified: FST-like 1 (FSTL1), IGFBP7 (FSTL2), FSTL3, FSTL4, and FSTL5. FSTL1 induces gene expression of various NF-*κ*B-related cytokines and chemokines and regulates NF-*κ*B signaling pathway expression, such as IL-1*β*, TNF-*α*, IL-6, CXCL8/IL-8, and CCL-2/monocytes and MCP-1 in macrophages [44]. FSTL1 can also induce pro-inflammatory responses by signaling IKKB-NF-*κ*B [45]. The sensor component of the NLR family pyrin domain containing 3 (NLRP3) inflammasome plays a crucial role in innate immunity and inflammation. Chen and Liu [46] also found that downregulation of FSTL-1 inhibits NLRP3 and TLR4/NF-*κ*B signaling pathways to reduce inflammatory damage during streptococcus pneumoniae infection. FSTL can modulate NF-*κ*B involved in inflammatory processes in reepithelialization, osteoarthritis, chondrocyte expression, and promotion of osteoclast formation in diabetic wound healing. FSTL1 stimulation can activate the phosphorylation of p65 [47]. The FSTL3 promoter contains an element of NF-*κ*B, which in turn stimulates FSTL3 expression [48]. Studies show that the levels of FST increased in NAFLD models, correlating with enhanced levels of collagen and TGF-*β*1, induced mitochondrial beta-oxidation, and downregulated fatty acid synthase activity [49]. FST is associated with adipose tissue IR and related features, and FST attenuates insulin-mediated suppression of lipolysis in adipocytes, thereby promoting NAFLD [50].

### 2.6. SHBG

SHBG (a homodimeric glycoprotein produced by hepatocytes) is closely related to metabolic abnormalities [51]. Both *in vivo* and *in vitro* experiments demonstrated that serum SHBG levels are reduced in NAFLD and that accumulation of hepatic fat rather than whole body fat is the determinant of decreased circulating SHBG expression [52]. Decreased SHBG may also be secondary to inflammatory factors, as increased TNF-*α* in response to JNK and NF-*κ*B activation reduces SHBG production [53]. It remains unclear that NF-*κ*B regulates the expression of human SHBG by altering its promoter activity [53]. SHBG plasma levels regulate hepatic lipogenesis via PPAR*γ* and are involved in IR in NAFLD patients [54]. SHBG is reduced in T2DM and is closely associated with the risk of NAFLD in patients with diabetes or polycystic ovary syndrome [55]. This shows that SHBG can be used as an important indicator for NAFLD. In addition, SHBG inhibits NAFLD-related hepatocarcinogenesis in postmenopausal women [56]. The above findings suggest that the role of SHBG in NAFLD-HCC may be related to the expression of steroid hormones, which complicates the interpretation of changes in SHBG levels and requires further exploration.

### 2.7. AHSG

Alpha 2-Heremans-Schmid glycoprotein (AHSG, also known as fetuin-A) is a hepatokine with a multifunctional glycoprotein structure [57]. Cumulative evidence points to a significant association between fetuin-A and NAFLD progression. ASHG is positively correlated with hepatic steatosis and is elevated in NAFLD. Furthermore, it can trigger IR in target tissues such as liver and skeletal muscle [58], and insulin sensitivity is significantly enhanced in ASHG-knockout mice [59]. ASHG is an endogenous inhibitor of tyrosine kinase that abrogates insulin downstream signaling, and serum-purified human ASHG has also been shown to significantly disrupt insulin-stimulated phosphorylation of IR and IRS-1 [60]. Meanwhile, earlier work also found that AHSG acts as an endogenous ligand for TLR4, which not only promotes lipid-induced IR, but also mediates the activation of TLR4 and NF-*κ*B pathways leading to an inflammatory cascade [61]. Reduction of ASHG/TLR4/JNK/NF-*κ*B pathway activation exerts a protective effect against inflammation and obesity as well as liver-related insulin resistance [62]. Some studies have shown that NF-*κ*B can regulate ASHG promoter activity [63]. Fatty acids significantly enhanced the expression of ASHG in the HepG2 by increasing the binding of NF-*κ*B to its promoter. Therefore, focusing on the crosstalk between NF-*κ*B and ASHG could be helpful in treating NAFLD.

### 2.8. LECT2

LECT2 is an obesity-related hepatokine. Clinical studies have shown that serum LECT2 expression is elevated in NAFLD patients. *In vitro* studies showed that inhibition of hepatic LECT2 expression in mice attenuated HFD-induced hepatic steatosis, whereas hepatic overexpression of LECT2 aggravated HFD-induced hepatic steatosis and inflammation [64]. LECT2 is positively correlated with inflammation and steatosis, and the increase of LECT2 converts residual hepatic macrophages into M1-like phenotypes and promotes the development of hepatic inflammation [65]. Shen et al. [66] found that LECT2 treatment upregulated the NF-*κ*B-binding activity and nuclear translocation of p65. They also found that Raf-1 in macrophages mediates LECT2 activation of NF-*κ*B, and LECT2-specific changes are responsive to Raf-1 phosphorylation [66]. Sterol-regulatory element binding protein-1c (SREBP-1c) is a novel insulin/JNK2-regulated gene, and the JNK2/SREBP-1c pathway mediates insulin-induced fatty acid synthesis. In addition, LECT2 can promote cellular lipid accumulation by promoting inflammatory markers through I*κ*B and NF-*κ*B phosphorylation and IL-6 expression, as well as by SREBP-1c-mediated signaling [67]. Thus, LECT2 promotes the development of NAFLD.

## 3. Stellakines and NF-*κ*B Activation

**Figure 2:**
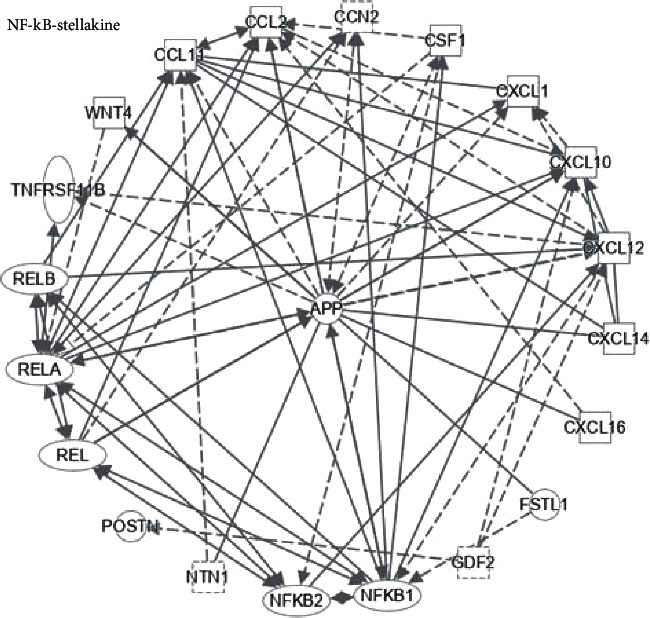
NF-*κ*B and stellakine. IPA showed the molecular pathways of stellakines and NF-*κ*B network. APP: amyloid precursor protein, CC chemokines (such as CCL2, CCL11), CSF1: colony stimulating factor 1 (also known as M-CSF), CTGF: connective tissue growth factor (also called CCN2), CXC chemokines (such as CXCL1, CXCL10, CXCL12, CXCL14, CXCL16), GAS6: growth arrest-specific 6, NTN1: netrin-1, POSTN: periostin. Solid lines indicate direct regulation; dashed lines indicate indirect regulation.

HSC activation is an important link in the progression of chronic liver diseases to liver cirrhosis and even liver cancer. Activated HSCs secrete numerous pro-inflammatory factors and produce ECM components such *α*-SMA and type I and type III collagen fibers, which drive liver inflammation and mediate hepatocyte death, thus promoting the development of liver disease [68]. Xiong et al. [69] found that activated HSCs in NASH mouse model not only produce ECM components, but also secrete multitudinous signal protein molecules, called “stellakines,” such as amyloid precursor protein (APP), CC chemokines (such as CCL2, CCL11), macrophage-colony stimulating factor (M-CSF, also known as CSF1), connective tissue growth factor (CTGF, also called CCN2), and CXC chemokines (such as CXCL1, CXCL10) (Figure 2).

### 3.1. CSF1 and POSTN

CSF1 is a myeloid cytokine released during infection and inflammation, which has also been shown to regulate the differentiation, proliferation, survival, and activation of monocytes and macrophages [70]. Mohallem and Aryal [71] have demonstrated that CSF1 is highly upregulated in TNF*α*-treated cells, suggesting that upregulation of CSF1 may activate pro-inflammatory responses and leukocyte invasion of adipose tissue through noncanonical activation of NF-*κ*B signaling mediated by the TNF receptor. The noncanonical NF-*κ*B, the p52-RelB dimer, is thought to respond to selective receptor signals that mediate adaptive immune functions. NIK (NF-*κ*B-inducing kinase) is a central component in the noncanonical NF-*κ*B signaling pathway [72]. Studies have also shown that the CSF1 receptor (CSF1R) also promotes the induction of noncanonical NF-*κ*B signaling during macrophage differentiation [73], which may have the potential to contribute to hepatic repair in fibrotic livers, as macrophage accumulation and phagocytic activity contribute significantly to this process [74]. POSTN (another stellakine) is mainly derived from activated HSCs, and its main function is regulating cell adhesion, proliferation, migration, and apoptosis [75, 76]. POSTN expression can be induced by NF-*κ*B and other pro-inflammatory transcription factors *in vitro* [77]. However, POSTN cooperates with TNF-*α* or IL-1*α* to activate NF-*κ*B in fibroblasts and then induces the expression of CCL2/MCP1, CCL4/MCP1, CCL7/MCP3, CXCL1/KC, CXCL2/MIP-1*α*, and IL-1*β* [78]. These results show that interaction between POSTN and NF-*κ*B activation may be a key factor in regulating the inflammatory pathogenic link of NAFLD.

### 3.2. NTNI, GAS6, and WNT4

Netrin-1 (NTN1) is included in a family of laminin-related secreted proteins. NTN1 regulates the activation of NF-*κ*B through the uncoordinated-phenotype-5A (also called UNC5A, a NTN1 receptor), because not only does the activation of UNC5A significantly activate the phosphorylation of NF-*κ*B/p65 at Ser536, but the UNC5A upregulates the expression of c-MYC (the NF-*κ*B downstream target) by activating NF-*κ*B to promote cell proliferation [79]. The RAC1 gene is involved in cellular growth and cell-cycle regulation. KCNQ1 opposite strand/antisense transcript 1 (KCNQ1OT1) is a long noncoding RNA gene. Interference or inhibition of NTN1-NF-*κ*B further inhibits c-MYC and leads to downregulation of the expression of RAC1 and inhibition of the hedgehog pathway via downregulation of KCNQ1OT1, ultimately inhibiting HSCs proliferation and epithelial-mesenchymal transition (EMT) in liver fibrosis [80].

Growth arrest-specific 6 (GAS6) is thought to be involved in the stimulation of cell proliferation. GAS6 inhibits NF-*κ*B phosphorylation, chemotaxis, and adhesion affinity between monocytes and endothelial cells [81]. GAS6 prevents activation of TNF receptor associated factor 6 (TRAF6) and NF-*κ*B by upregulating suppressor of cytokine signaling 3 (SOCS3) in cellular models [82]. AS6-AS2 is an identified cancer-related lncRNA. TLR ligands reduce GAS6 by downregulating GAS6-AS2 expression via NF-*κ*B activation, suggesting a bidirectional feedback system between GAS6 and inflammation [81]. WNT4 inhibits NF-*κ*B by noncanonical WNT signaling [83]. WNT4 also activates *β*-catenin/NF-*κ*B by the cooperation of frizzled 4 and low-density lipoprotein receptor-related protein 6 (LRP6) signaling [84]. Recombinant WNT4 proteins potently inhibit the apical step of transforming growth factor beta-activated kinase 1 phosphorylation, as well as the subsequent steps of RANKL (RANKL induces each critical step in NF-*κ*B activation) -induced p65 phosphorylation and I*κ*B*α* phosphorylation and degradation [83,85]. Furthermore, WNT4 also inhibits NF-*κ*B-dependent transcription [83]. These facts further demonstrate that several genes interact with NF-*κ*B to regulate the development and progression of NAFLD.

### 3.3. CXCL1, CXCR4/7, CXCL12, and CXCL16

CXC chemokines are a chemokine subfamily with a CXC motif at the N-terminus, also known as *α*-chemokines. 17 CXC chemokines have been reported (such as CXCL1, CXCL10, CXCL12, CXCL14, and CXCL16). CXC chemokines affect tumorigenesis on cell recruitment, aggregation migration, invasion, angiogenesis, angiostasis, and lymphangiogenesis [86]. CXCL1 has an additional ELR motif, and VEGF activity induces increased expression of CXCL1 in endothelial cells, which induces angiogenesis through CXCR2 (receptor of CXCL1) [87, 88]. Activation of HIF-1 and NF-*κ*B increases CXCR2 expression in cancer cells during chronic hypoxia [89]. In HUVECs, NF-*κ*B/p65 increases the expression of long intergenic non-protein-coding RNA 1693 (LINC01693, which functions as a miRNA-302d sponge) to enhance CXCL12 expression [90]. miRNA-302d decreases the expression of CXCL12, which shows that hypoxia increases the expression of CXCL12 through NF-*κ*B [86]. Mallory–Denk bodies formed in human alcoholic hepatitis and NASH can mediate TLR3/4 signaling through the NF-*κ*B-CXCR4/7 pathway [91]. LPS and NF-*κ*B activation increase the expression of CXCL16 in HUVEC [92]. Silencing of CXCL16 expression decreases tumor cell migration and cell proliferation via the reduction of NF-*κ*B activation [93].

### 3.4. CCL11, CCL2, CXCL10, and CTGF

CC chemokines are a subfamily of 27 chemokines that are involved in intercellular communication and can also regulate the microenvironment in some tumors [94]. Activation of ERK MAPK by CCL11 mediates apoptosis resistance in cancer cells [95]. CCL11-CCR3 interaction activates phosphatidylinositol 3-OH kinase/serine-threonine kinase (PI3K/Akt) signaling pathway in endothelial cells to induce angiogenesis [96]. Furthermore, activation of the CCR3 receptor by CCL11 increases the expression of VEGF in HCC, thereby indirectly promoting angiogenesis [97]. It has been reported that macrophages with NF-*κ*B-mediated mixed canonical and STAT-6-mediated alternating activation phenotypes can produce CCL11 [98]. This makes us think that NF-*κ*B regulation and activation of CCL11 can promote cancer angiogenesis and affect the prognosis of HCC. p50 can inhibit liver inflammation and immune response by regulating CCL2 and CXCL10 secretion by HSCs, alleviating liver injury [99]. p65 inhibits the activation of HSCs and reduces the secretion of stellakines to improve the inflammatory response [100]. Tumor cell-derived CTGF transmits growth-promoting signals to HCC cells by activating nearby HSCs, so CTGF has also been identified as a cornerstone in the HCC microenvironment, but anti-CTGF antibodies are susceptible to inhibiting this interaction [101]. CTGF promotes the nuclear accumulation of p50 and p65 to protect the survival of the primary HSCs. Chaqour et al. [102] demonstrated that NF-*κ*B binds to the binding site in the promoter region of CTGF. Intranuclear translocation of NF-*κ*B via the Smad-independent pathway can regulate CTGF expression, whereas TGF-*β*-induced CTGF expression is inhibited by Bay 11-7082 (NF-*κ*B inhibitor) [103].

## 4. Adipokines and NF-*κ*B Activation

**Figure 3:**
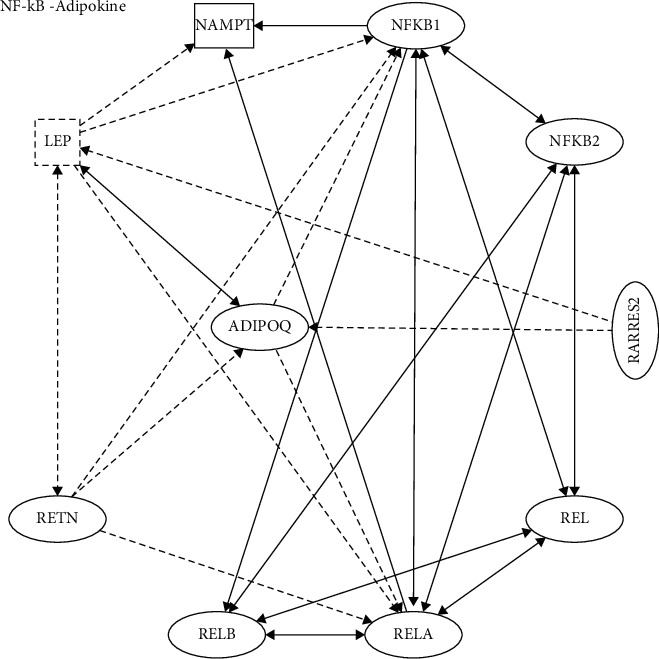
NF-*κ*B and adipokines. IPA showed the molecular pathways of stellakines and NF-*κ*B networks. LEP: leptin, ADIPOQ: adiponectin, RETN: resistin, NAMPT: nicotinamide phosphoribosyl transferase. Solid lines indicate direct regulation; dashed lines indicate indirect regulation.

Adipose tissue (both subcutaneous and visceral) is a fundamental player in systemic inflammatory processes associated with obesity. The involvement of adipose tissue in communication between metabolically active organs requires mediators such as cytokines and adipokines [104]. Adipokines are involved in the regulation of appetite and satiety (e.g., leptin), fat distribution, insulin secretion and sensitivity (e.g., leptin and adiponectin (ADIPOQ)), glucose metabolism (e.g., leptin, adiponectin), adipogenesis, and lipid metabolism and also in the accumulation of endothelial dysfunction and inflammation in the liver (e.g., TNF-*α*) [105,106]. This implicates that adipokines as important factors in regulating human metabolic diseases such as obesity and NAFLD. Several important adipokines such as leptin, ADIPOQ, RETN, and nicotinamide phosphoribosyl transferase (NAMPT) are closely related to NF-*κ*B (Figure 3).

### 4.1. RETN

Resistin (RETN) is co-secreted with adiponectin in white mouse adipocytes. NF-*κ*B plays an important role in resistin (RETN) -induced bone remodeling, hyperglycemia-induced increase in RETN expression [107], *in vivo* development of IR, stimulation of pro-inflammatory cytokines in macrophages and peripheral blood mononuclear cells, and endothelial dysfunction. A study showed that NF-*κ*B can regulate gene expression of inflammatory responses, cytokines [108], and related oxidative-responsive adipokines such as TNF-*α* [109].

Kumar et al. [110] demonstrated that two closely linked SNPs underlie the genetic regulation of RETN expression in human monocytes, as they control NF-*κ*B1-p50/p50-homodimer binding, histone acetylation, and C-methylation of the RETN promoter. Regarding RETN's role in inflammatory responses, it upregulates the expression of monocyte chemoattractant protein 1 (MCP-1) and the release of vascular cell adhesion molecule 1 and intracellular adhesion molecule 1 (ICAM-1) and activates NF-*κ*B in endothelial cells [111,112]. RETN-stimulated phosphorylation and subsequent activation of the signaling proteins p38, JNK, and ERK constitute another mechanism for pro-inflammatory cytokine production [113]. Inhibition of the NF-*κ*B transcription factor may limit the effect of adipokines on joint injury, as visfatin and RETN activate NF-*κ*B signaling through phosphorylation of the ERK/p38/MAPK pathway that induces pro-inflammatory and OA-degrading processes [114]. RETN originally described as an adipocyte-specific hormone is an important link between obesity, insulin resistance, and diabetes. RETN may also act by regulating the canonical NF-*κ*B pathway [115]. Another study also confirmed that human RETN increased p65 expression in a dose-dependent manner, thereby promoting keratin 8 transcriptional expression to inhibit glycogen accumulation in HepG2 cells [116]. Finally, RETN was found to have a pro-inflammatory role in HSCs, suggesting that RETN is involved in liver fibrosis [117]; the serum levels of RETN were higher in NAFLD and were positively correlated with liver inflammation [118].

### 4.2. LEP

Leptin (LEP), a 16-kDa protein containing 167 amino acids and discovered in 1994, is secreted from adipocytes. LEP is mainly involved in metabolic regulation through three signaling pathways: the Janus kinase-signal transducer and activator of transcription (JAK-STAT) pathway, PI3K/AKT pathway, and extracellular signal-regulated kinase (ERK) pathway [119].

It was found that NF-*κ*B activation in osteoarthritis (OA) patients upregulates two factors (LEP and its receptor Ob-R) produced by articular cartilage [120]. Chen et al. suggested that LEP could accelerate the expression of NF-*κ*B/p65(RELA), and the crosstalk between the NF-*κ*B signaling pathway and the JAK/STAT signaling pathway could increase the secretion of pro-inflammatory cytokines (e.g., human granulocyte macrophage-colony stimulating factor, IL-1*α*, and IL-6) [121]. Faustmann et al. [122] found that NF-*κ*B/p65 activation was inversely correlated with LEP concentrations. Moreover, RNA-seq analysis by Reilly et al. [123] found that NF-*κ*B1 deficiency could aberrantly regulate the expression of inflammatory effectors, antigen presentation, and immune checkpoints through JAK-STAT signaling. Recent studies have shown that inhibition of microglia can reduce food intake, reduce diet-induced obesity, and improve LEP signaling by an NF-*κ*B-dependent approach [124]. Rian, a small RNA, could regulate numerous target genes, and the human homologs of some of these microRNAs have been proposed to both stimulate and inhibit tumorigenesis in other types of malignancies. Rian/miR-210-3p/NF-*κ*B1 (the target of miR-210-3p) feedback loop inactivates the PI3K/Akt pathway [125], suggesting a direct link between NF-*κ*B1 and PIK3/Akt pathway. In general, the role of LEP and NF-*κ*B is reciprocal; LEP can directly activate NF-*κ*B signaling, while NF-*κ*B can indirectly regulate LEP through JAK-STAT, PI3K/AKT, and ERK signaling pathways. LEP may regulate pyroptotic-like death of macrophages and hepatocytes in NAFLD progression via CD8+ T lymphocytes [126]. Taken together, LEP and NF-*κ*B may communicate as promising candidates for the treatment of inflammatory and metabolic diseases.

### 4.3. ADIPOQ

Adiponectin, also known as ADIPOQ, discovered in 1995, is a 30-kDa protein consisting of 244 amino acids, which mainly signals on target cells through two adiponectin receptors, ADIPOQ receptor 1 (AdipoR1) and AdipoR2 [127]. Binding of adiponectin to AdipoR1 and R2 activates the phosphorylation of AMPK (downstream mediator of ADIPOQ) and p38 MAPK, as well as increasing PPAR*α* ligand activity [128]. The main physiological function of ADIPOQ is to increase insulin sensitivity, promoting anti-fibrotic and anti-inflammatory effects (switching the macrophage phenotype to an anti-inflammatory state reduces inflammatory initiation by reducing Toll-like expression receptor 4), enhancing insulin secretion and vasculoprotective and anti-atherosclerotic effects, and regulating food intake and energy expenditure [128].

ADIPOQ effect on NF-*κ*B activation is bidirectional in different monocyte cell lines, both stimulating and inhibiting it [129]. It was previously reported that ADIPOQ increases NF-*κ*B p65 and enhances the production of pro-inflammatory factor IL-6 through the AdipoR1-AMPK-p38-NF-*κ*B pathways in human synovial fibroblasts [130]. Furthermore, ADIPOQ inhibits NF-*κ*B activation and suppresses inflammation by regulating AMPK [131], which is supported by the finding that ADIPOQ inhibits microglial inflammatory response to amyloid-*β* oligomer through AdipoR1-AMPK-NF-*κ*B signaling [130]. Yang et al. [132] show that ADIPOQ exerts anti-inflammatory and anti-oxidative stress effects through the neuronatin (NNAT)/NF-*κ*B pathway in adipocytes. ADIPOQ can also protect skeletal muscle by reducing the activity of NF-*κ*B in muscle inflammation and myogenesis [133]. Furthermore, ADIPOQ proved to be a biomarker of NAFLD progression to steatohepatitis [134].

### 4.4. NAMPT

Nicotinamide phosphoribosyltransferase (NAMPT) is associated with aging and diabetes. NAMPT modulates cellular metabolism by affecting the activity of nicotinamide adenine dinucleotide (NAD)-dependent enzymes through the biosynthetic activity of NAD, an essential coenzyme involved in cellular redox reactions and a substrate for NAD-dependent enzymes [135]. In addition to its biological enzymatic functions, NAMPT can also affect a variety of physiological processes and participate in metabolic regulation. Glucose and oxidized low-density lipoprotein activate the PI3K-AKT pathway to stimulate NAMPT protein expression and release in human adipocytes [136]. *In vitro* studies have shown that NAMPT is stimulated by IR-inducing factors (IL-6 and TNF-*α*) [137] and that its mRNA expression is increased during adipogenesis. These facts suggest that NAMPT can affect glucose metabolism, IR, and lipogenesis. Several studies have reported that extracellular NAMPT exerts pro-inflammatory effects through induction of iNOS, activation of ERK1/2 [138] and NF-*κ*B [139,140], and cytokine production [140].

Activation of PPAR*α* downregulates NAMPT expression in the liver of NAFLD patients [141]. In contrast, NAMPT has an antiapoptotic effect in NAFLD, as apoptosis in stress-exposed rat hepatocytes can be ameliorated by its overexpression [141]. NAMPT regulates pro-inflammatory cytokine production and inhibits insulin signaling resistance through JAK2/STAT3 and IKK/NF-*κ*B signaling in HepG2 cells [142]. Furthermore, NAMPT induces NF-*κ*B and MAP kinase (MKK) 3/6-p38 signaling to increase CC chemokine ligand 20 (also called CCL20, an important cytokine for inflammation and fibrosis in the liver) expression in macrophages, which suggests that NAMPT can promote the activation of fibrotic markers in HSCs [143], which may be an important therapeutic target for the treatment of NAFLD.

Taken together, the findings suggest that adipokines regulate the processes involved in the pathogenesis of NAFLD by regulation of oxidative stress, apoptosis, lipid and glucose metabolism, inflammation, and IR [135]. The adipokines-NF-*κ*B network seems to be mainly involved in the inflammatory and fibrosis processes in NAFLD, and whether it is involved in other physiological processes needs further study. Moreover, adipokines have negative effects on NAFLD or NASH, such as (1) activation of HSCs (ADIPOQ, LEP, and RETN); (2) recruitment of macrophages (ADIPOQ and NAMPT); and (3) activation of monocytes (ADIPOQ and RARRES2).

## 5. Myokines and NF-*κ*B Activation

**Figure 4:**
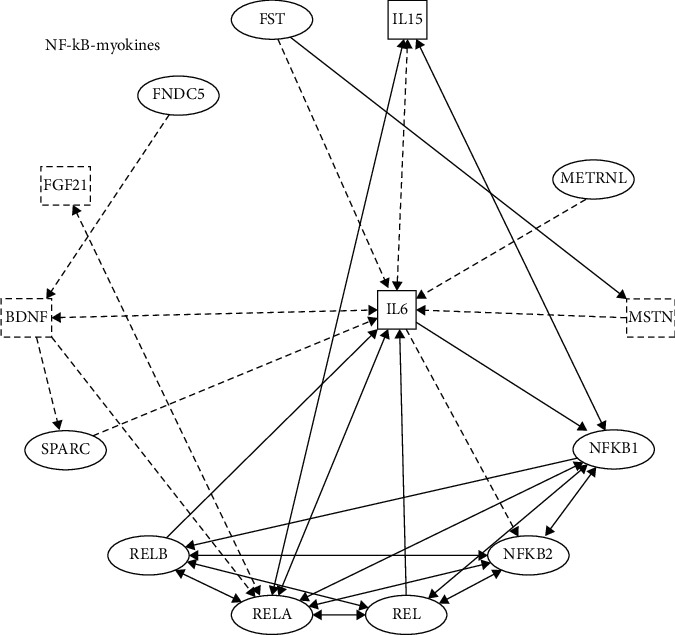
NF-*κ*B and myokines. IPA showed the molecular pathways of myokines and NF-*κ*B. IL-6: interleukin-6, IL-15: interleukin-15, BDNF: brain-derived neurotrophic factor. Solid lines indicate direct regulation; dashed lines indicate indirect regulation.

Actin is a cytokine or peptide produced by skeletal muscle cells and released into the circulation, acting on other cells, tissues, or organs through autocrine, paracrine, or endocrine effects [144,145]. There are many types of myokines, but only 650 myokines have been confirmed so far. Myokines primarily mediate intramuscular signaling and muscle-organ crosstalk with the brain, liver, adipose tissue, gut, pancreas, bone, vascular bed, and skin during exercise [146,147]. IPA assay showed that interleukin-6 (IL-6), interleukin-15 (IL-15), FGF21 (described in 1.1), and brain-derived neurotrophic factor (BDNF) are involved in the development of NAFLD through NF-*κ*B (Figure 4).

### 5.1. IL-6

Multiple cell types, including leukocytes, adipocytes, and myocytes, are known to secrete IL-6 [148]. IL-6 is associated with chronic inflammation in diseases such as obesity and T2DM [146]. IL-6 promotes insulin-stimulated glucose uptake, lipolysis, and fat oxidation [149]. NAFLD is associated with the elevation of IL-6 [150]. Increased concentrations of soluble IL-6 receptor *α* (sIL-6R*α*) and gp130/sIL-6R*β* (both in the IL-6 cytokine family) prevent NAFLD progression in obese patients [151]. Steatosis leads to upstream activation of IKK-*β* (inhibitor of NF-*κ*B) to increase signaling of the transcription factor NF-*κ*B, which induces pro-inflammatory mediators such as TNF-*α*, IL-6 [152], and IL-1*β* production. These cytokines could recruit and activate the Kupffer cells [153] to mediate inflammation in NASH [154], RELB [155], NF-*κ*B/p65 [156], and NF-*κ*B2 [157]. In conclusion, it is feasible that NF-*κ*B can affect NAFLD and its related complications by regulating IL-6 to regulate inflammation, lipid metabolism, fatty acid oxidation, and insulin sensitivity.

### 5.2. IL-15

IL-15 is a member of IL-2 superfamily, and its main function is to regulate anabolism in skeletal muscle. It can also mediate exercise-induced muscle-fat crosstalk [158], as it downregulates lipid accumulation in preadipocytes, reduces white adipose tissue (WAT) mass by stimulating adiponectin secretion, and counteracts the effect of TNF-*α* on muscle protein degradation [159]. In addition, IL-15 treatment prevents HFD-stimulated fatty liver [160], which has the potential to delay the progression of NAFLD.

Decreased hepatic resident memory CD8+ T (CD8+ Trm) cells maintained by IL-15 in tissues delayed fibrosis regression, and adoptive transfer of these cells protected mice from fibrotic progression [161]. Furthermore, IL-15 mediates HFD-induced lipid accumulation and inflammation promoting NAFLD [162]. NF-*κ*B is a major mediator of IL-15 signaling in brain endothelial cells, through which IL-15 affects cellular permeability, endocytosis, and intracellular trafficking at the blood-brain barrier level, and it also induces phosphorylation of p65 and nuclear translocation [163]. p50 deficiency may accelerate NASH progression to fibrosis by promoting IL-15 activation to stimulate natural killer T (NKT) cell recruitment [164].

### 5.3. BDNF

BDNF is a member of the neurotrophic family that is widely expressed in the adult brain and is mainly involved in immune-inflammatory responses [165]. NK-*κ*B can regulate BDNF promoter activity [165]. Xu et al. [165] found that BDNF expression appears to require activity of the MyD88/NF-*κ*B signaling pathway to induce innate immune responses which also induces innate immune responses [165]. BDNF is an important marker for preventing and treating NAFLD [166], as its serum level has positive association with NAFLD [167]. However, no *in vitro* or *in vivo* experiments have found the signaling pathway between BNDF and NAFLD.

### 5.4. New Management Measures for NAFLD and NASH

Different region- and country-specific factors influence the epidemiological incidence of NAFLD. Experts provide new insights into NAFLD disease burden, screening, diagnosis and referral patterns, and available treatment options for NAFLD and NASH in the Middle East [168]. The prevalence of obesity, MetS, and T2DM is increasing simultaneously, and the high prevalence of these NAFLD risk factors increases the disease burden of NAFLD. Given the high prevalence of NAFLD in high-risk populations, most international guidelines recommend routine liver enzyme screening, ultrasonography, and transient elastography for these patients [169,170]. The American Association for the Study of Liver Diseases (AASLD) guidelines currently do not recommend routine screening for NAFLD because of uncertainty about the performance characteristics of diagnostic tests and available treatment options, and they recommend routine screening for NAFLD patients suspected of having diabetes [1]. In contrast, European guidelines recommend screening for NAFLD in all patients with steatosis with persistent abnormalities in liver enzymes [170]. Noninvasive diagnostic methods including serum biomarkers, routine radiology (e.g., ultrasound), computed tomography, magnetic resonance imaging, and assessment of liver stiffness using transient elastography (FibroScan®) and magnetic resonance elastography may be useful for NAFLD/NASH. Diagnosis plays a supporting role [171]. Liver biopsy is considered the gold standard for NASH and fibrosis staging [172]. If a patient is suspected of having NASH tendencies during clinical diagnosis and treatment, timely referral and multidisciplinary team management should be performed. Sanai et al. [168] performed noninvasive examinations and fibrosis scores in NAFLD patients with elevated liver enzymes and radiographic steatosis, respectively. They classified the examination results into low-risk, intermediate-risk, and high-risk. Diet and exercise therapy for patients with low-risk scores, liver biopsy for patients with intermediate scores, and dietary therapy for patients with NAFLD with high-risk scores when laboratory therapy is not appropriate. However, an effective diagnosis of NAFLD is currently lacking [173], and effective multidisciplinary collaboration across healthcare fields would be beneficial to improve the understanding of NAFLD and NASH.

## 6. Mechanism of Action and Therapeutic Benefits of Silymarin

Silymarin is a lipophilic extract that can be extracted from the dried seeds and fruits of the milk thistle plant (*S. marianum*). Johannes Gottfried Rademacher first discovered in the 19th century that an extract or “tincture” of milk thistle seeds was beneficial in treating patients with liver disease [174,175]. Silymarin (milk thistle extract) is a complex mixture of compounds of plant origin, mainly identified as flavonoid lignans, flavonoids (taxifolin, quercetin), and polyphenolic molecules [176]. The four major flavonolignan isomers in silymarin are silybin, isosilybin, silymarin, and silybin. Silymarin has antioxidant properties, anti-inflammatory properties, anti-fibrotic effects, and insulin resistance modulation.

German scientists at the University of Munich first isolated silymarin in 1968, and then it was described and patented by Madaus (German herbal medicine manufacturer) as a specific treatment “for liver diseases” [175]. Rottapharm/Madaus (Cologne, Germany) developed the first commercial silymarin formulation which meets the analytical specifications reported in European Pharmacopoeia 01/2005 “Milk Thistle Fruit” [177]. In 2000, the National Center for Complementary and Alternative Medicine (NCCAM) published a review indicating that the clinical efficacy of milk thistle has not been established, despite evidence of silymarin in various liver conditions [178]. From November 2012 to August 2014, at a tertiary hospital in Kuala Lumpur, Malaysia, patients were randomly assigned to receive silymarin (700 mg; *n* = 49 patients) or placebo (*n* = 50 patients) three times a day for 48 weeks [179]. The results showed that the percentage of patients achieving the primary efficacy outcome did not differ significantly between the groups (32.7% in the silymarin group and 26.0% in the placebo group; *P* = 0.467) [179]. However, patients in the silymarin group had less fibrosis, liver stiffness measurements (30% or more; 24.2%), mean aspartate aminotransferase to platelet ratio index, fibrosis-4 score, and NAFLD fibrosis score. Between January 2017 and October 2017, 90 patients with NAFLD, who were followed up in the Department of Hepatology and Gastroenterology at the University of Campania “Luigi Vanvitelli,” were randomized into two groups: treatment group (silibinin plus vitamins D and E, *n* = 60) and untreated group (control group, *n* = 30) [180]. Their results showed that NAFLD patients in the treatment group had statistically significant improvements in metabolic markers, oxidative stress, endothelial dysfunction, and disease progression (*p* < 0.05) after 6-month treatment [180]. From January 2014 to June 2014, 62 patients with chronic HCV decompensated cirrhosis were randomized according to treatment plan at Cairo University Hospital: group A (*n* = 31) received 1,050 mg/day silymarin; group B (*n* = 31) received 420 mg/day silymarin [181]. From October 2016 to April 2017, Anushiravani et al. [182] conducted a randomized, double-blind, placebo-controlled trial of 150 consecutive NAFLD patients from an outpatient clinic in southern Iran: lifestyle plus placebo treatment (*n* = 30), metformin 500 mg/day (*n* = 30), silymarin 140 mg/day (*n* = 30), pioglitazone 15 mg/day (*n* = 30), and vitamin E 400 IU/day (*n* = 30) for 3 months. The results showed that after only 3 months, silymarin significantly improved liver transaminases, waist circumference, and BMI in NAFLD patients without any specific side effects [182]. In 2019, a trial tested a proprietary standardized formulation of silymarin (Legalon®, Rottapharm|Madaus, Mylan) at 5 medical centers in the United States [183]. Legalon® 420 mg, 700 mg treatment, or placebo given 3 times daily for 48 weeks. After 48 weeks, there were no significant differences in adverse events between treatment groups. After changing doses, the results were the same [183]. They considered a possible reason was that a large number of participants (49, 63%) did not meet histological entry criteria. Patients initially diagnosed with hepatitis C infection were collected from Bakhtawar Amin Medical College and Hospital in Multan, Pakistan, and randomized into two groups: the control group (*n* = 15) received direct-acting antivirals (DAA) alone (sofosbuvir and ribavirin; 400 mg/800 mg daily); the treatment group (*n* = 15) did not use DAA but received adjuvant therapy with silymarin (400 mg/day) and DAA (400/800 mg/day) over an 8-week period [184]. The results showed that adjuvant silymarin treatment increased the efficiency of DAA; decreased ALT, alkaline phosphatase (ALP), AST, and bilirubin levels (*p* > 0.05); improved superoxide dismutase (SOD) and total antioxidant status (TAS); reduced and oxidized glutathione (GSH and GSSG) and malondialdehyde (MDA) (*p* < 0.05); and reduced latent viral load, which suggests that silymarin may have a unique role in alleviating hepatitis C [184]. Since these clinical trials have demonstrated beneficial effects of silymarin in liver diseases such as NAFLD/NASH, it is gradually being registered as a drug for the treatment of liver diseases in many countries in Europe, Asia, America, Africa, and Australia. We then reviewed the possible mechanisms by which silymarin regulates NAFLD/NASH.

Silymarin increases the production of glutathione in the liver by increasing the availability of cysteine, its biosynthetic substrate, which in turn helps to enhance its antioxidant capacity in liver tissue [185]. The main mechanisms of silymarin in protecting liver cells are as follows: (1) It stabilizes membrane permeability by inhibiting lipid peroxidation and helps the liver maintain glutathione levels [185]. (2) Silymarin also blocks the activation of NF-*κ*B by inhibiting the production of TNF-*α*, interferon-*γ*, IL-2, and IL-4, thereby preventing various effects of toxic chemicals such as carbon tetrachloride [186–188]. (3) It also inhibits the expression of TNF-*α* induced by *α*-amanita toxin of the poisonous mushroom [189]. Silymarin has a weak inhibitory effect on the formation of PGE2 in isolated rat Kupffer cells but has a strong inhibitory effect on the formation of LTB4, even at a low concentration (15 *μ*mol/l) [190]. Selective inhibition of the latter explains the anti-inflammatory potential of silymarin in Kupffer cells. The anti-fibrotic effect of silymarin has been demonstrated in an animal model of alcohol-induced liver fibrosis in nonhuman primates treated with chronic alcohol [191]. Silymarin significantly reduces alcohol-induced increases in type I hepatic collagen [191]. Silibinin improves IR in a rat model of NAFLD by reducing visceral adiposity, enhancing lipolysis, and inhibiting gluconeogenesis [192]. Due to current lifestyle changes, both the frequency and amount of alcohol consumption are increasing, and silymarin may reduce lipid peroxidation and cell necrosis by enhancing cell viability [193]. Butorova et al. [194] treated patients with NAFLD or NASH with diet or silymarin for 2 months and showed that silymarin reduced or normalized parameters of liver function (transaminases levels) and improved ultrasound parameters of liver anatomy. Several recent randomized clinical studies have shown that silymarin significantly reduces the severity of steatosis, hepatic ballooning, and fibrosis compared with placebo, which may help improve liver conditions affected by NAFLD [195]. Compared with placebo, silymarin treatment reduces alanine aminotransferase (ALT) and aspartate aminotransferase (AST) levels, which indicates that silymarin improves liver function [196,197]. In addition, in clinical trials in patients with liver cirrhosis, silymarin exerted antioxidant effects by inducing lipid peroxidation (as a free radical scavenger) and affecting enzyme systems associated with cellular damage leading to fibrosis and cirrhosis, which significantly reduce liver-related death [198]. Silymarin may also exert hepatoprotective effects by reducing oxidative stress and cytotoxicity. Silymarin, a Eurosil 85®-derived preparation, has great therapeutic potential and has been developed for the treatment of NAFLD and NASH [133, 179, 199]. Phase III clinical trials have confirmed that silymarin is the best drug for NAFLD patients at present, but its standardization, dosage form, and dosage regimen are difficult problems that need to be overcome now [195]. Taken together, these data suggest that silymarin treatment is effective in reducing fibrosis, liver stiffness, and metabolic markers (ALT, AST) levels; improving oxidative stress and endothelial dysfunction; and delaying disease progression in different regions. Silymarin is expected to be an effective emerging therapeutic drug for NAFLD and NASH.

## 7. Conclusions

In this review, the current signaling pathways in which hepatokines, stellakines, adipokines, myokines, and NF-*κ*B are mutually regulated are discussed to explore their impact on inflammation, liver injury, and fibrosis in the progression of NAFLD. The review focuses on novel regulatory mechanisms and modes of action with known or suspected roles in regulating NAFLD and NASH metabolism (Figures 1–4).

Our study found that activation of NF-*κ*B signaling pathway by hepatokines, stellakines, adipokines, and myokines aggravates liver injury and inflammation, promotes the progression of NAFLD to HCC, and regulates the proliferation, invasion, and metastasis of HCC. FGF21 knockdown promotes the progression of NASH to HCC through the TLR4/NF-*κ*B/IL-17A axis. FGF21 downregulates I*κ*B*α* phosphorylation and NF-*κ*B nuclear translocation to inhibit HSC activation, reducing the ratio of Bcl-2 to Bax to promote apoptosis to improve fibrosis. Elevation of NF-*κ*B by HMGB1 accelerates the early liver injury and inflammatory response of NAFLD, promotes the progression of NAFLD to HCC, and regulates cell proliferation, invasion, and metastasis of HCC cell lines. Sitagliptin can protect the liver by directly inhibiting the proliferation of activated HSCs by promoting Nrf2 and inhibiting the NF-*κ*B pathway, reducing the expression levels of inflammatory and pro-fibrotic factors. On the other hand, sitagliptin ameliorates NAFLD inflammation by reducing HMGB1-mediated TLR4/NF-*κ*B signaling. However, TLR3/4 also mediates the NF-*κ*B-CXCR4/7 pathway in the formation of Mallory–Denk bodies in human alcoholic hepatitis and NASH. p50 suppresses liver inflammation and immune response and alleviates liver injury by regulating HSC secretion of CCL2 and CXCL10. p65 ameliorates the inflammatory response by inhibiting HSC activation and reducing the secretion of stellakines. CTGF promotes the nuclear accumulation of p50 and p65 to protect the survival of primary HSCs, and CTGF can also transmit growth-promoting signals to HCC cells by activating nearby HSCs. Furthermore, NAMPT induces NF-*κ*B and MKK3/6-p38 signaling to increase CCL20 in macrophages to promote fibrosis in HSCs. As a myokine, IL-6 is significantly associated with the increased risk of NAFLD and promotes HSC activation. p50 deficiency may promote IL-15 activation to stimulate NKT cell recruitment, which accelerates the progression of NASH to fibrosis.

Inhibiting the activation of NF-*κ*B may represent a promising emerging class of NAFLD or NASH therapeutics. However, the therapeutic potential of hepatokines, adipokines, and myokines has not been effectively confirmed in clinical practice, so more research is needed to explore the mysteries among them. In the next step, we will carry out *in vitro* and *in vivo* experiments to explore the signaling pathway that inhibits NF-*κ*B in regulating NAFLD, which will be beneficial to the development new molecular treatments for NAFLD.

We should improve NAFLD education and awareness; manage NAFLD risk factors and comorbidities; establish appropriate screening, assessment, and diagnostic measures; and promptly refer to hepatologists when predisposition to NASH is identified. Furthermore, some emerging drugs such as silymarin have great therapeutic potential for NAFLD/NASH.
